# Exploring an energy-density adjustment to the Nutri-Score algorithm for general foods

**DOI:** 10.1007/s00394-026-04105-5

**Published:** 2026-09-19

**Authors:** Anna Amberntsson, Mari Mohn Paulsen, Marta Angela Bianchi, Bryndis Eva Birgisdottir, Anja Pia Biltoft-Jensen, Dina Moxness Konglevoll, Anne Lise Brantsaeter, Lene Frost Andersen, Marianne Hope Abel

**Affiliations:** 1 Department of Food Safety, Norwegian Institute of Public Health, Oslo, Norway; 2 Centre for Sustainable Diets, Norwegian Institute of Public Health, Oslo, Norway; 3 Department of Agriculture and Food, RISE Research Institutes of Sweden, Gothenburg, Sweden; 4 Unit for Nutrition Research, Faculty of Food Science and Nutrition, School of Health Sciences, University of Iceland, Reykjavík, Iceland; 5 National Food Institute, Technical University of Denmark, Lundtofte, Denmark; 6 Department of Nutrition, Institute of Basic Medical Sciences, University of Oslo, Oslo, Norway; 7 Department of Physical Health and Ageing, Norwegian Institute of Public Health, P.O. Box 222, NO-0213 Skoyen, Oslo, Norway; 8 Centre for Evaluation of Public Health Measures, Norwegian Institute of Public Health, P.O. Box 222, NO-0213 Skoyen, Oslo, Norway

**Keywords:** Nutrient profiling, Nutri-Score, Reference units, Proof‑of‑concept evaluation, Algorithm modification

## Abstract

**Purpose:**

The Nutri-Score categorizes foods by nutritional value per 100 g or 100 ml, which may lead to poor discriminatory ability for products with low energy density and large portion sizes, like ready-to-eat meals. We hypothesized that adapting the Nutri-Score algorithm to account for variation in energy density could better capture nutritional quality, particularly in lower energy-dense foods like ready meals, without substantially affecting other food scores. The aim of this study was to perform a proof‑of‑concept evaluation of adapting the Nutri-Score algorithm for general foods to different levels of energy density.

**Methods:**

This study utilized the Norwegian food databases Tradesolution and Unil (*N* = 25,813) to compare the energy-adjusted algorithm with the Nutri-Score 2023.

**Results:**

The energy-adjusted Nutri-Score shifted food categorizations for 22% of products. Foods with low energy density, such as ready meals, showed increased scoring variation, indicating a more nuanced nutritional quality assessment, as intended. In these foods, we also saw a decline in favourable scores and an increase in unfavourable ones. However, high-energy-density food categories, like cakes and pastries, more often received more favourable scores. Also, the discriminatory ability between low-fat and full-fat products was reduced.

**Conclusion:**

While the energy-adjusted Nutri-Score improved scoring differentiation for certain low-energy-density foods, it showed unfavourable side-effects, indicating that overall, it is not superior to the Nutri-Score 2023. Nonetheless, adapting the algorithm to energy density offers potential for improved nutrient profiling methodologies in specific contexts. Further refinement of the weighting system could address observed weaknesses, optimizing this approach for broader application.

## Introduction

Nutrient profiling is a scientific method used to classify food and beverages based on their nutritional composition [1]. It involves analysing the levels of various nutritional components in a food and comparing them with established criteria or benchmarks [2]. Different nutrient profiling systems use different reference units for calculation of a summary score. Most common are scoring by weight or volume, such as per 100 g or 100 mL (e.g., the Nutri-Score [3]), scoring by energy, such as per 100 kcal (e.g., the Food Compass [4]), or scoring by portion or serving size (e.g., the Nutrient Rich Foods Index [5]). The Nutri-Score is in use as a front-of-pack nutrition label in several European countries and provides simplified and summarized information of the nutritional content. The Nutri-Score has three separate algorithms; one for beverages [6], one for general foods, and one for high-fat foods like fats, oils, nuts, and seeds [7]. The algorithms are adapted to the markedly different energy densities between these food groups. However, the general foods-algorithm covers a wide range of energy densities, from bouillon soup to mayonnaise. Thus, the algorithm for general foods might not be optimal for products in the higher or lower range of energy density and fail to capture meaningful variation in nutritional quality in those foods [8, 9]. In fact, the Nutri-Score has been criticized for being “confounded by water content” [4] since the scoring per 100 g is much affected by the water content of a product. This disadvantage is greater for nutrient profiles scoring across-the-board (i.e. using the same algorithm for a wide range of foods) compared with category-based profiles because the energy density is more variable between food categories than within [10].

Scoring of components by energy may provide a more comparable assessment across foods and beverages than by weight [11, 12]. However, foods with low energy density might receive disproportionately high scores for negligible nutrient concentrations [11]. The only method directly linked to the amount of food consumed is the scoring of components based on per serving or portion [11]. Using per serving or portion considers the food’s contribution to the overall dietary balance. However, there is currently no uniform standard for serving or portion sizes established at the EU level for different food categories. It has been argued that using 100 kcal as the reference unit provides a more accurate measure of the nutrient density of favourable nutrients, as nutritionally favourable foods appear more nutrient-dense per calorie consumed, while using 100 g as the reference unit is more suitable for unfavourable nutrients because it correlates strongly with the energy density of the foods [11]. The feasibility of combining reference units has been shown in the French SAIN, LIM system, which scores favourable components per 100 kcal and unfavourable components per 100 g [13].

Within the NewTools project [14], the Nutri-Score has previously been evaluated in a Norwegian setting [8, 15, 16] and some revisions have been proposed to improve the algorithms and to enhance the performance of this scoring system from a Nordic perspective [9]. A potential limitation of the Nutri-Score 2023 identified in the NewTools project is the tendency for ready meals and mixed dishes to cluster within a narrow range of Nutri-Score categories, particularly category C [8, 9]. This pattern may indicate limited discrimination of nutritional characteristics within this product group, although it could also reflect relatively similar nutrient compositions among ready meals. The high water content and relatively low energy density in many ready-to-eat dishes, compared with single-ingredient foods, make it difficult for the algorithm to capture meaningful variation in nutrient content. This limitation is a weakness of concern since the portion sizes of ready meals are often large. Consequently, it has been hypothesised that the Nutri-Score 2023 may provide limited guidance in distinguishing between more and less nutritionally favourable ready meals.

We hypothesized that adapting the Nutri-Score algorithm to account for variation in energy density could improve the discrimination in nutritional quality of products with low energy density, like ready meals and mixed dishes, without causing major changes in scores of other foods. The aim of this study was to explore the performance of the Nutri-Score algorithm for general foods in capturing differences in nutritional quality across varying energy densities of food, and to perform a proof‑of‑concept evaluation of adapting the Nutri-Score algorithm for general foods to different levels of energy density. The modified algorithm was compared to the Nutri-Score 2023 by assessing changes in food scores across food categories in a dataset of pre-packed foods sold in Norway.

## Methods

This study encompasses an assessment of the discriminatory ability of the Nutri-Score 2023 algorithm for general foods across different levels of energy density, as well as the development and proof-of-concept evaluation of a proposed modified algorithm adapted to four energy-density levels. The scoring with the modified algorithm was evaluated against the Nutri-Score algorithm published in 2023 [7]. Both the Nutri-Score 2023 and the modified algorithm were applied in two Norwegian food databases.

### Food databases

Tradesolution is a comprehensive database that includes most pre-packed foods sold in Norway. The database is owned by three retail group companies and Grocery Suppliers of Norway (DLF Norway). Unil is a private label food supply company owned by the Norwegian retail group NorgesGruppen and their products are included in a separate database. For this study, the sample of foods consisted of all items in Tradesolution (version June 2023, *N* = 28,691 food items) and Unil (version November 2022, *N* = 577 food items) that met the criteria for calculating the Nutri-Score 2023 main algorithm for general foods [17]. After items with missing information for Nutri-Score components (except for fibre and fruits, vegetables, and legumes (FVL)), duplicates, and products with implausible values, the databases were merged and consisted of 25,833 unique food items.

As dietary fibre is not mandatory in the nutritional declaration within the European Union, products with missing information on fibre (*n* = 14,929) were assigned 0 points for fibre in the algorithms. Neither database contained information on FVL content, which is a key component of the Nutri-Score. All products were assigned zero points for FVL (%) in the algorithms except for foods classified as fruits, berries, vegetables, and legumes (*n* = 1,615), which were all assigned a value of > 80% for calculation of the Nutri-Score.

### The Nutri-Score main algorithm for general foods

We applied the most recent update of the Nutri-Score main algorithm for general foods from 2023 [7] to items in the food databases. The main algorithm for general foods includes all solid foods, soups and stocks, with the exclusion of fats, oils, nuts and seeds. Nutri-Score is calculated per 100 g or 100 mL using the unfavourable components; energy (kJ), salt (g), saturated fat (g), and total sugars (g), and the favourable components; protein (g), dietary fibre (g), and percentage of FVL.

### Adapting the scoring across varying energy densities of foods

Energy-density adjustment refers to the overall modification of the Nutri-Score algorithm to account for differences in energy density between foods. In the present study, this adjustment was implemented through energy-density-based weighting, whereby nutrient component scores were multiplied by weighting factors determined by the food’s energy-density level.

**Fig. 1:**
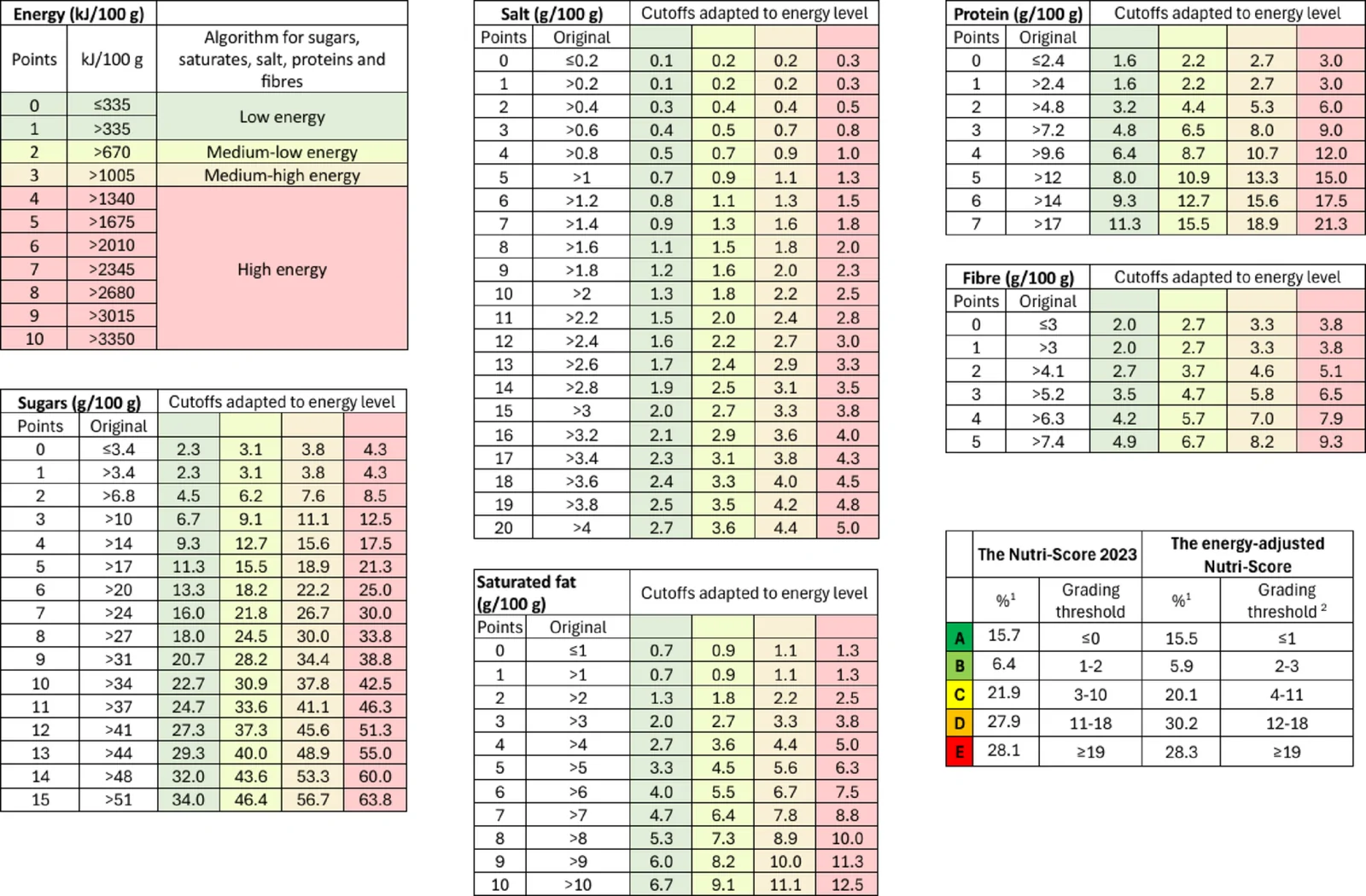
New cut-offs for the proposed energy-adjusted Nutri-Score algorithm

The proposed method for adapting the scoring of components across varying energy densities of foods has been independently devised by our research team. We propose to balance the scoring of nutrient content both by 100 g and by 100 kcal with 50% each for all components in the Nutri-Score except for the energy and FVL component, since the energy component is the basis for the weighting factors and the FVL component uses a different point allocation than the other components. To create the modified Nutri-Score algorithm for general foods, we applied weighting factors reflecting the energy-density level (i.e. the points for energy in the Nutri-Score 2023) of each food item for each component in the score (Table 1). The median energy density among all general food items in the food database was 1 MJ/100 g, and therefore, this level was chosen as the reference point where the Nutri-Score algorithm should remain unchanged (weight = 1). A weighting factor for each energy-density level was determined to balance the scoring of nutrient content by 100 g and by 100 kcal. The Nutri-Score 2023 energy component for general foods assigns 0–10 points in 335 kJ increments per 100 g (0 points for ≤ 335 kJ/100 g; 10 points for > 3350 kJ/100 g, Table 1). In general, weights were defined for each energy-density level so that the content of nutritional component X per 100 g and per 100 kcal would equally determine the points given for nutritional component X. Initially, the energy-adjusted algorithm included eleven distinct energy-density levels, one for each of the energy-density levels (0–10 points) in the Nutri-Score 2023 algorithm. During preliminary testing, we observed that the highest and lowest weighting factors produced disproportionate effects on the scoring outputs. Weighting factors > 1.5 (corresponding to very low-energy-density foods) resulted in overly large penalties or rewards for minimal nutrient concentrations. Conversely, weights < 0.8 (corresponding to high-energy-density foods) led to implausibly favourable scores in categories such as cakes and sweet snacks. To avoid these distortions, we reduced the original eleven energy-density levels into four and these are presented in Table 1. Thus, the weighting factors were capped, which resulted in four weighting levels (1.5, 1.1, 0.9 and 0.8). The final weighting scheme was therefore determined empirically based on the observed behaviour of the algorithm and intended to avoid implausible scoring outcomes at the lowest and highest energy-density levels. The resulting attributing points for each nutritional component of the energy-adjusted Nutri-Score is presented in Fig. 1. As the purpose of the study was to conduct a proof-of-concept test of the proposed methodology, we limited the analyses to a single weighting structure.

**Table 1:** Suggested weighting factors for the scoring of components in the Nutri-Score 2023 algorithm by energy-density level

Energy-density level (points for energy)	Energy-density (MJ/100g)^a^	Crude weighting factor for equal weighting of content per 100 g and per MJ^b^	Weighting factors – capped to avoid disproportional weight on content per MJ
0	≤ 0.335	3.5	1.5
1	> 0.335	1.5	1.5
2	> 0.670	1.1	1.1
3	> 1.005	0.9	0.9
4	> 1.340	0.8	0.8
5	> 1.675	0.8	0.8
6	> 2.010	0.7	0.8
7	> 2.345	0.7	0.8
8	> 2.680	0.7	0.8
9	> 3.015	0.7	0.8
10	> 3.350	0.6	0.8

Energy bands follow the Nutri‑Score energy thresholds (335 kJ increments)

^a^The cut-off for the energy component in the Nutri-Score 2023

^b^The weighting factors for each energy-density level was calculated for the mid-value of energy density for each energy level (i.e., 0.335 MJ/2 for level 0 etc.)

1. Distribution of foods in Tradesolution and Unil databases (*N* = 25 833 pre-packed foods).
2. Cutoffs were adapted to keep a similar distribution of scores and compensate for the energy-adjusted algorithm assigning more points in total to foods.

As this work was intended as a proof-of-concept test of the proposed methodology, we limited the analysis to applying equal weighting (50% each) to nutrient content per 100 g and per 100 kcal and therefore did not explore alternative weighting schemes. The new total score is calculated in the same way as the Nutri-Score 2023 main algorithm for general foods [7]. To calculate the total score, the points for the unfavourable components are summed. If the sum of the unfavourable component is ≥ 11 points, then the following formula is applied:

$$\begin{aligned} Nutri - Score\;points = &\,total\;unfavourable\;points \\ & - \;points\;from\;fibre \\ & - \;points\;from\;FVL. \\ \end{aligned} $$

If the sum of the unfavourable component is < 11 points or the food is cheese, then apply the following formula:

$$ \begin{aligned} Nutri - Score\;points = & \;total\;unfavourable\;points \\ & - \;total\;favourable\;points. \\ \end{aligned} $$

Finally, the thresholds for A–E were adjusted to match the current distribution of the Nutri-Score in the dataset (Fig. 1).

### Ethics

Ethical approval was not required as no human or animal subjects were involved in this study.

### Statistical analyses

The distribution of the Nutri-Score (A-E) with the Nutri-Score 2023 and the proposed energy-adjusted Nutri-Score was compared through cross-classification and percentage agreement. Variation in the Nutri-Score 2023 and the energy-adjusted Nutri-Score across varying energy densities of foods was visualized through boxplots. The distributions (in percent) were presented for the Nutri-Score 2023 and the energy-adjusted version in all foods as well as within selected sub-categories with varying energy densities (cheese, yoghurt, red meat, fish, and ready meals). As the dataset comprised the majority of pre-packed foods available on the Norwegian market, analyses were considered descriptive rather than inferential, and no confidence intervals or significance testing were performed. Algorithm development was conducted using Microsoft Excel (version 2308 Build 16.0.16731.20542). Evaluation was performed using Stata (StataCorp Stata Statistical Software: Release 17, College Station, TX, USA). Figures were created in RStudio 2025.05.1 + 513 “Mariposa Orchid” Release.

## Results

In total, 22% (*n* = 5,647) of all products in the databases received a different Nutri-Score (A-E) with the energy-adjusted version of the Nutri-Score 2023 (Table 2). A larger proportion of products moved to a more unfavourable score (12%, *n* = 3,155) than to a more favourable score (10%, *n* = 2,492). Among products receiving a different Nutri-Score, most (95%) were moved one score class up or down, but some products moved several classes.

**Table 2:** Classification of food products (n (%)) in the Tradesolution and Unil databases (*N* = 25,813) according to the Nutri-Score 2023 and the current energy-adjusted Nutri-Score

The Nutri-Score 2023	The energy-adjusted Nutri-Score					
	A	B	C	D	E	Total
A	3,705 (91.5)	254 (6.3) ↓	57 (1.4) ↓↓	33 (0.8) ↓↓↓	0	4,049 (100%)
B	276 (16.8) ↑	909 (55.2)	320 (19.4) ↓	141 (8.6) ↓↓	0	1,646 (100%)
C	19 (0.3) ↑↑	354 (6.3) ↑	3,985 (70.5)	1,290 (22.8) ↓	5 (0.1) ↓↓	5,653 (100%)
D	0	11 (0.2) ↑↑	819 (11.4) ↑	5,324 (74.0)	1,045 (14.5) ↓	7,199 (100%)
E	0	0	0	1,013 (13.9) ↑	6,253 (86.1)	7,266 (100%)

Arrows illustrate the direction and magnitude of changes in Nutri-Score between the Nutri-Score 2023 and the energy-adjusted Nutri-Score. Arrow down indicates products obtaining a more unfavourable Nutri-Score, and arrow up indicates obtaining a more favourable Nutri-Score. Single arrow indicates moving one class. Double arrow indicates moving two classes. Tripple arrow indicates moving three classes.

### Variation in Nutri-Score and energy-adjusted Nutri-Score over energy density

**Fig. 2:**
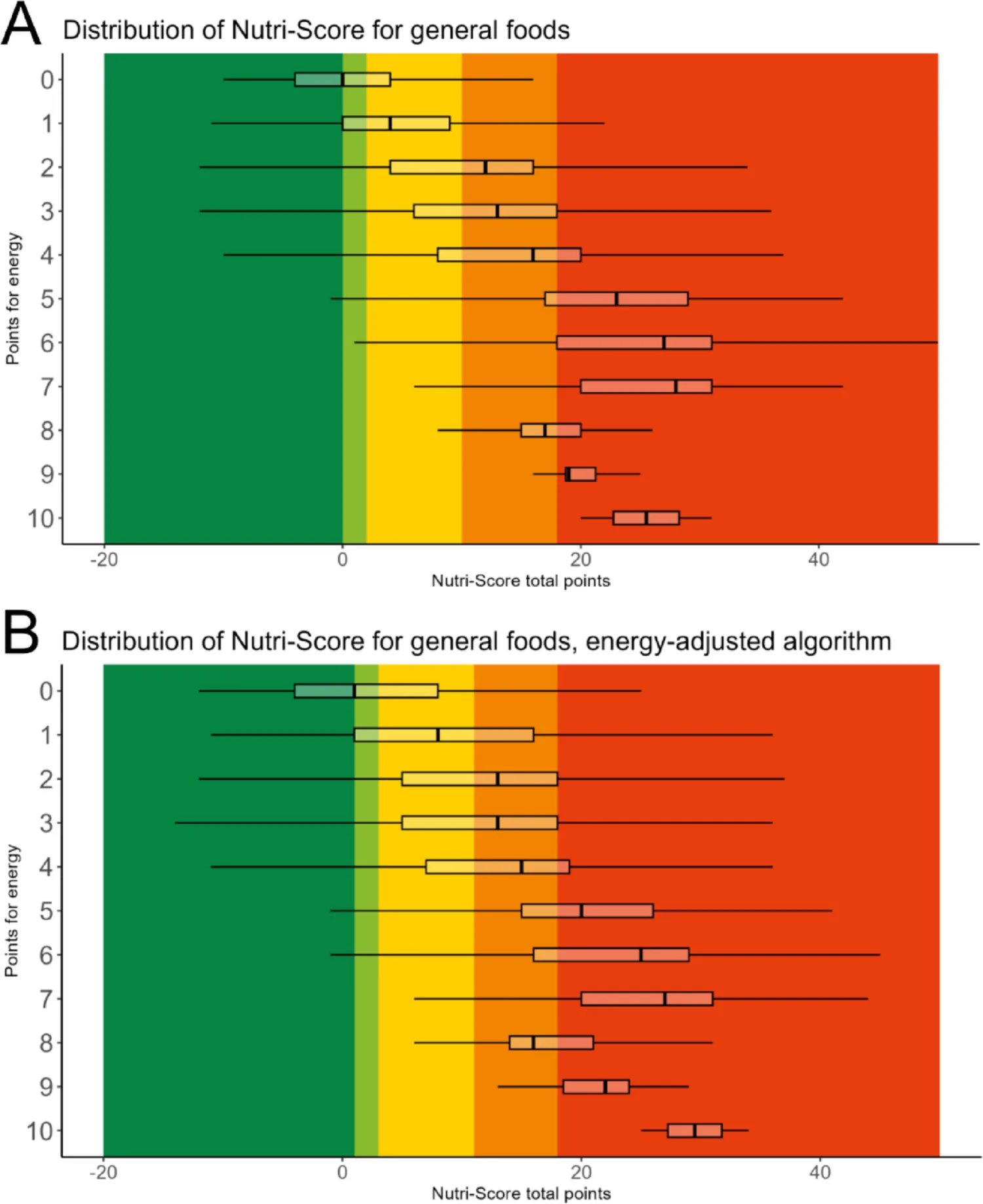
Variation in Nutri-Score over the range of energy density in the Nutri-Score 2023 (a) and the energy-adjusted version of the Nutri-Score (b) in Tradesolution and Unil databases (*N* = 25,813). The coloured area represents Nutri-Score (A-E). Products with lower energy density (fewer points for energy) tend to get a better Nutri-Score with the 2023 algorithm compared with products of higher energy density. The energy-adjusted Nutri-Score showed an increased score variation, particularly in foods with low energy density, and a slight shift in the distribution of median Nutri-Score points towards more unfavourable scores

**Fig. 3:**
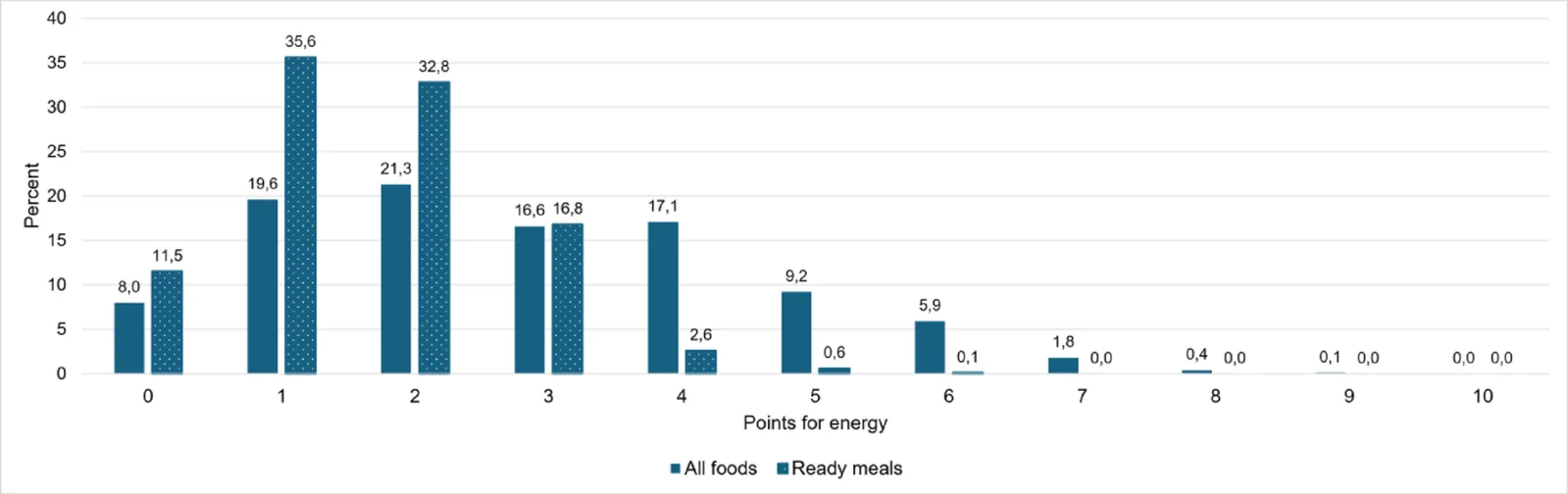
Percentage of all foods (*n* = 25,813) and among ready meals (*n* = 1,466) by points for energy in Tradesolution and Unil databases. Compared with the overall distribution of points for energy in all foods, ready meals were clustering at 1 and 2 points for energy

The scoring of products over the range of energy density shows that products with lower energy density almost exclusively obtained a favourable Nutri-Score (i.e., A to C) with the 2023 algorithm, and products of high energy density (i.e., receiving 4 points or more for energy) obtained almost all a D or E (Fig. 2a). Products in the highest level of energy density all obtained a poor score (E), however, only 18 products (< 0.01%) had ≥ 9 points for energy in the current datasets (Fig. 3).

The energy-adjusted Nutri-Score showed an increased score variation, particularly in foods with low energy density (Fig. 2b). There was also a slight shift in the distribution of median Nutri-Score points with the energy-adjusted algorithm towards more unfavourable scores, which was partly balanced by the change in thresholds for the A-E classification (Fig. 1).

### Variation in Nutri-Score and energy-adjusted Nutri-Score by food categories

#### Ready meals

**Fig. 4:**
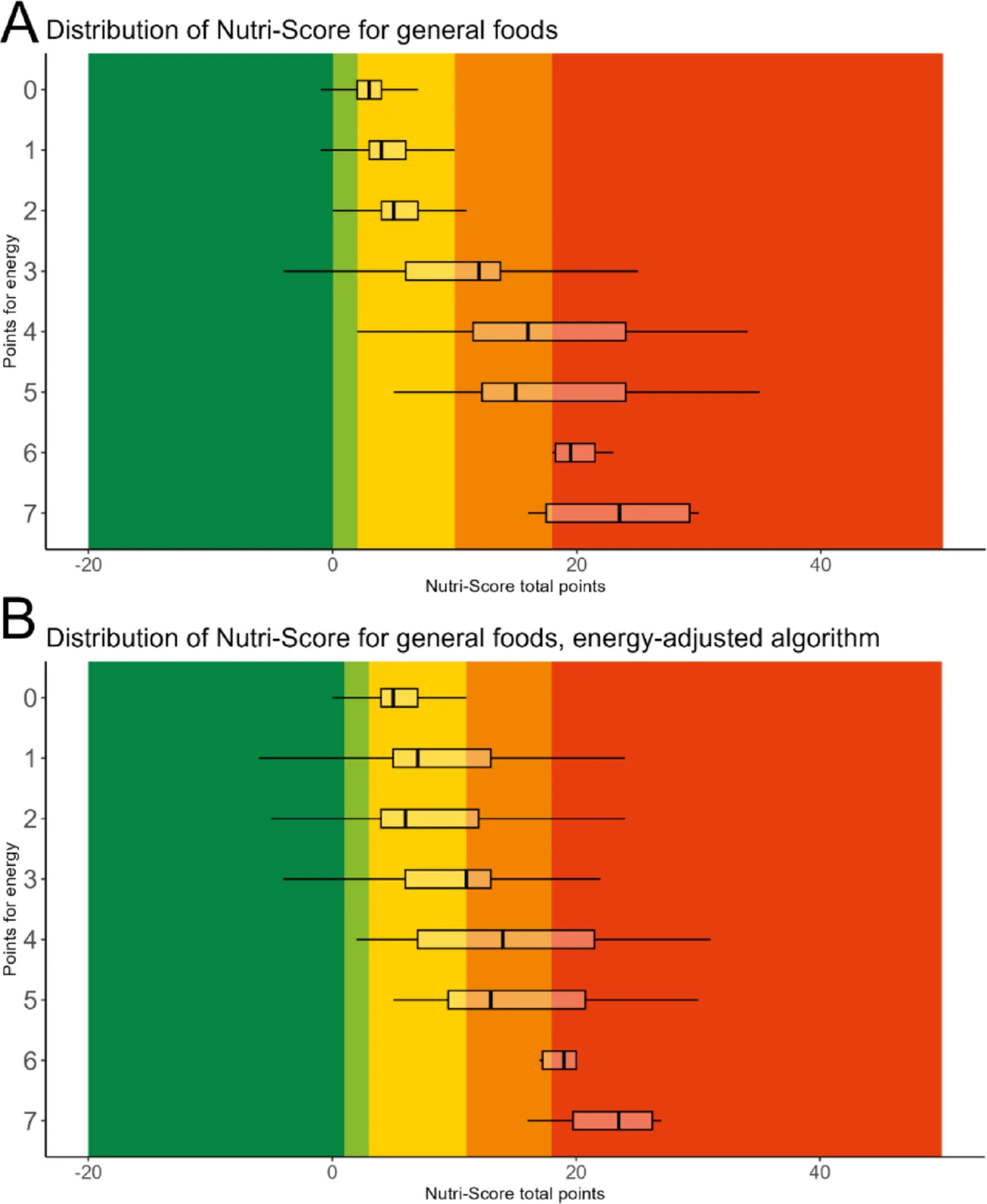
Variation in Nutri-Score in ready meals (*n* = 1,466) over the range of energy density in the Nutri-Score 2023 (a) and the energy-adjusted version of the Nutri-Score (b) in Tradesolution and Unil databases. The coloured area represents Nutri-Score (A-E). There was little variation in scores among ready meals with the Nutri-Score 2023 algorithm, and most ready meals with low energy density (≤ 2 points for energy) received Nutri-Score C. The energy-adjusted algorithm increased the variation in scores for ready meals, especially in ready meals with low energy density. There was also a slight shift in the distribution of Nutri-Score points with the energy-adjusted algorithm towards an overall poorer score for ready meals

In contrast to the overall distribution of points for energy, ready meals were clustering at 1 and 2 points for energy (69% of all ready meals, *n* = 1,014, Fig. 3). Score variation among ready meals was limited with the Nutri-Score 2023 algorithm (Fig. 4a). Most ready meals with low energy density (≤ 2 points for energy) received Nutri-Score C. Thus, most ready meals with low energy density received a favourable or neutral Nutri-Score. The energy-adjusted algorithm increased the variation in scores for ready meals, especially in ready meals with low energy density (Fig. 4b). There was also a slight shift in the distribution of Nutri-Score points with the energy-adjusted algorithm towards an overall poorer score for ready meals (Fig. 4b; Table 3).

**Table 3:** The Nutri-Score 2023 and energy-adjusted Nutri-Score in the Tradesolution and Unil databases (*N* = 25,813)

Food category	*n*	The Nutri-Score 2023 %					The energy-adjusted Nutri-Score %					Changed Nutri-Score %
		A	B	C	D	E	A	B	C	D	E	
Plant-based meat	208	24	13	35	26	2	19	18	31	29	3	35
Mayonnaise-based salads	198	0	3	42	49	6	0	2	69	26	3	35
Meat spread	793	1	2	15	33	49	1	1	2	35	61	32
Plant-based yoghurt	67	13	36	49	2	0	24	25	30	21	0	30
Fish	2,603	36	10	14	20	20	33	6	8	26	27	30
Biscuits, cakes and pastries	579	0	2	23	43	32	1	3	34	44	18	30
Sauce, dressings, dips	1,214	2	3	29	37	29	2	2	24	36	36	29
Ready meals	1,466	5	12	60	20	3	6	10	53	26	5	29
Breakfast cereals	206	27	13	36	19	5	36	11	39	11	3	28
Vegetables, legumes	1,278	60	7	16	14	3	55	5	18	13	9	26
Plant based cheese	40	7	8	5	22	58	12	8	0	7	73	25
Plant-based sandwich spread	44	0	9	45	46	0	0	14	41	34	11	25
Potatoes	191	14	28	52	6	0	25	16	42	17	0	24
Salty snacks	516	1	1	9	64	25	1	0	22	62	15	23
Jam	503	4	3	25	65	3	5	3	5	84	3	22
Bread	1,698	15	17	55	11	2	18	21	52	8	1	21
Cheese	991	1	0	11	70	18	1	0	15	59	25	18
Red meat	5,748	13	3	17	35	32	11	3	16	34	36	18
Sweet snacks and desserts	5,000	2	1	9	23	65	2	2	9	32	55	18
Poultry	422	51	8	10	30	1	49	8	5	35	3	16
Fruits, berries	333	38	5	29	26	2	38	3	32	25	2	16
Yoghurt	255	28	21	47	2	2	33	14	43	8	2	14
Plain cereals, grains	1,196	41	21	22	8	8	45	20	20	8	7	13
Sweet sandwich toppings	158	1	0	8	21	70	1	2	9	21	67	9
Egg	121	95	3	1	1	0	97	1	0	2	0	5

Among ready meals with ≤ 670 kJ/100 g (soups, stews, porridge, pasta dishes, and vegetarian ready meals), products were mainly moved from Nutri-Score B to C and from C to D (Table 4). Ready meals with 671–1,005 kJ/100 g (mainly pizza, sandwiches, sushi, dumplings, and ready meals with meat) had increased variance in scores and products moved from Nutri-Score C to B or D. Ready meals with 1,006–1,340 kJ/100 g and those with > 1340 kJ/100 g were mainly pizza, sandwiches, pies, and wraps. These products moved for the most part from Nutri-Score C to D.

#### Other food categories

The energy-adjustment affected foods across all food categories (Table 3). The food categories with the largest change in scoring with the energy-adjusted Nutri-Score were plant-based meat, mayonnaise-based salads, meat spread, plant-based yoghurt, fish, and biscuits, cakes and pastries. An increase in products receiving favourable Nutri-Scores of A and B, coupled with a reduction in those with less favourable scores of D and E, was observed in several categories. These included: plant-based sandwich spread, plain cereals and grains, bread, breakfast cereals, biscuits, cakes and pastries, sweet snacks and desserts, and sweet sandwich toppings. Conversely, other categories exhibited a decline in products achieving Nutri-Scores A and B, alongside an increase in products rated with D and E. These included: dairy yoghurt, red meat, meat spread, fish, vegetables and legumes, potatoes, sauce, dressings and dips, and ready meals.

Table 4 presents the energy-adjusted Nutri-Score across selected food categories with different energy density.

**Table 4:** The Nutri-Score 2023 and energy-adjusted Nutri-Score within selected food categories with varying energy densities in the Tradesolution and Unil databases (*N* = 25,813)

Food category	*n*	Nutri-Score 2023 %					Energy-adjusted Nutri-Score %					Changed Nutri-Score %
		A	B	C	D	E	A	B	C	D	E	
*Cheese*												
< 18% fat	125	7	0	55	31	7	6	1	48	32	13	26
18–29% fat	474	0	0	7	69	24	1	0	18	57	25	17
> 29% fat	392	0	0	1	84	15	0	0	1	70	29	17
*Yoghurt*												
≤ 3% fat, < 6% sugar	62	95	3	2	0	0	96	2	2	0	0	2
≤ 3% fat, ≥ 6% sugar	74	18	55	27	0	0	32	34	34	0	0	24
> 3% fat, < 6% sugar	21	0	48	52	0	0	0	43	38	19	0	24
> 3% fat, ≥ 6% sugar	98	0	2	89	5	4	0	2	78	16	4	11
*Red meat*												
< 10% fat, < 2 g salt	1,508	48	12	31	9	0	42	11	20	26	1	31
< 10% fat, ≥ 2 g salt	505	0	0	5	63	32	0	0	0	22	78	51
≥ 10% fat, < 2 g salt	2,457	1	0	25	63	11	1	1	25	64	9	15
≥ 10% fat, ≥ 2 g salt	2,072	0	0	0	13	87	0	0	0	8	92	8
*Fresh fish*												
< 2% fat, ≤ 0.3 g salt	304	100	0	0	0	0	100	0	0	0	0	0
< 2% fat, > 0.3 g salt	134	47	19	11	22	1	22	7	5	54	12	63
2–6% fat, ≤ 0.3 g salt	76	100	0	0	0	0	100	0	0	0	0	0
2–6% fat, > 0.3 g salt	36	19	53	6	19	3	17	3	3	69	8	67
> 6% fat, ≤ 0.3 g salt	180	98	1	0	1	0	98	1	1	0	0	2
> 6% fat, > 0.3 g salt	56	7	20	14	38	21	7	21	5	34	32	27
*Ready meals*												
≤ 670 kJ/100 g	729	6	19	69	4	2	7	11	53	25	4	35
671-1,005 kJ/100 g	468	4	8	66	21	1	5	13	56	24	2	17
1,006 − 1,340 kJ/100 g	234	2	2	31	59	6	2	6	53	33	6	32
> 1340 kJ/100 g	55	0	3	9	44	44	0	6	27	27	40	33

In the cheese category, products with 18–29% fat content demonstrated a shift towards more favourable Nutri-Scores, increasing the proportion with a C rating while reducing those with a D. Conversely, cheeses with less than 18% fat mainly transitioned from Nutri-Score C to D, while cheeses with > 29% fat were more frequently moved from D to E.

Regarding yoghurt, only one product among those with ≤ 3% fat and < 6% sugar content changed its Nutri-Score distribution from B to A. In contrast, yoghurts with ≤ 3% fat and ≥ 6% sugar demonstrated an increased proportion of products receiving Nutri-Scores A and C instead of B, increasing the variation based on content of saturated fat, sugar and protein. Notably, yoghurts containing > 3% fat received predominantly poorer Nutri-Scores regardless of sugar content, and were mainly moved from C to D.

Red meat products with < 10% fat and < 2 g salt generally moved towards less favourable scores across all Nutri-Score categories. Among meats with < 10% fat and ≥ 2 g salt, there was a transition from Nutri-Score C to D and from D to E. For red meat products with ≥ 10% fat and < 2 g salt, scores shifted predominantly from C and D to D and E, but also the other way – from D to C and E to D. Products with ≥ 10% fat and ≥ 2 g salt exhibited scoring changes mostly from D to E, although some transitions occurred in the opposite direction, from E to D.

For fish products, those containing > 0.3 g salt shifted towards less favourable scores across all Nutri-Score categories, regardless of fat content. Most of these fish products now scored D and some were even moved to an E. For example, cod with < 2% fat and 1.6 g salt/100 g, which previously was assigned with Nutri-Score A, was now moved to Nutri-Score D. Fish products with ≤ 0.3 g salt remained largely unchanged in their scoring, regardless of fat content.

## Discussion

This study aimed to explore the performance of the Nutri-Score algorithm for general foods in capturing differences in nutritional quality across varying energy densities of food. We found that products with lower energy density generally received a more favourable Nutri-Score than products with higher energy density, regardless of nutritional quality. Products in the lower end of energy density only rarely obtained a Nutri-Score D or E, whereas products in the higher end almost exclusively obtained a D or E. Among ready meals, products with low energy density commonly clustered at Nutri-Score C. Ready meals with low energy density typically received a favourable or neutral Nutri-Score. Ideally, Nutri-Score classification should primarily reflect nutritional quality regardless of energy density. As an example, an olive oil-based dressing with only olive oil and herbs should not be bound to get an E, and a ready meal which provides your whole daily maximum intake of salt in one portion should not be bound to get a C due to their energy-density levels. To potentially increase the variation in scores in products in the lower range of energy density, like ready meals, we proposed adapting the Nutri-Score 2023 main algorithm for general foods to four different levels of energy density by weighting the components in the score according to energy density. Overall, the energy-adjusted Nutri-Score showed a slight shift in the distribution of median Nutri-Score points and increased variation in scores in products with lower energy density. However, this adapted version also had some unfavourable side-effects. For example, the unhealthy food categories biscuits, cakes and pastries, and sweet snacks and desserts, obtained overall more favourable scores. Additionally, the scoring of low-fat alternatives, for example of cheese, was more heavily penalized for unfavourable components like salt compared to the full-fat version, and thus, the scoring would no longer consistently guide towards products with lower energy and saturated fat content. Thus, the energy-adjusted Nutri-Score did not outperform the Nutri-Score 2023 overall.

To the best of our knowledge, no previous study has attempted to adapt an across-the-board nutrient profiling system like the Nutri-Score to energy-density levels. However, the Nutri-Score 2023 has three different algorithms adapted to the very different energy density of ‘beverages’ and ‘oils, fats, nuts and seeds’ compared with other foods. Our research highlights a key challenge in nutrient profiling systems that predominantly use weight or volume-based scoring, namely the disproportionate favourability towards foods with lower energy density [11]. As observed, the Nutri-Score 2023 algorithm frequently assigns favourable scores to these products although the nutritional quality may be poor. As an example, ready meals, including several pizzas and other ready-to-eat dinners, typically cluster around Nutri-Score C. This tendency underscores a potential bias in evaluating nutritional quality, where water content plays a prominent role, despite not necessarily influencing the nutritional value. The large proportion of ready meals classified as Nutri-Score C may suggest limited discrimination within this food category. However, the present study did not assess whether these products differ substantially in nutritional quality according to independent measures.

In the current study, we found limited discriminatory performance of the Nutri-Score within ready meals and mixed dishes, with a significant majority (60%) of these products clustering at Nutri-Score C. Only 3% of ready-meals were assigned with Nutri-Score E. Whether this reflects an overestimation of nutritional quality or simply similarities in nutrient composition within these products remains uncertain. Few previous studies have evaluated the performance of the Nutri-Score 2023 within ready meals and mixed dishes. A study analysing a Greek branded food composition database revealed that over 70% of ready meals were rated as either Nutri-Score A or B using the 2023 algorithm [17]. Due to limited previous research, it remains unknown if the clustering of ready meals at Nutri-Score C is a context-specific issue rather than a universal challenge across all European markets.

The introduction of an energy-adjusted Nutri-Score algorithm attempted to address this limitation by recalibrating the scoring adapted to four different levels of energy density. This approach aimed to provide a more nuanced differentiation in nutrient profiles across diverse food categories, especially for products with low energy density, such as ready meals. Our results indicated increased variability in scores, particularly within ready meals. Whether this corresponds to a more accurate assessment of nutritional quality remains to be established. The observed shift where foods with higher energy density were less penalized for unfavourable contents, raises concerns. Unhealthy food categories such as biscuits, cakes and pastries, sweet snacks and desserts, and sweet sandwich toppings received overall more favourable scores, while healthier food categories such as dairy yoghurt, fish, vegetables and legumes, and potatoes received less favourable scores. Despite enabling greater score variation, the energy-adjusted model appeared to lead to inconsistencies affecting credibility across nutritional assessments for various food categories that align poorly with the dietary guidelines. Thus, the energy-adjusted Nutri-Score may provide better discrimination than the Nutri-Score 2023 for ready meals and mixed dishes but is not suitable as an across-the-board nutrient profile in its current form. There have been nutrient profiling models developed specifically to assess the overall nutritional value of dishes and meals [18, 19], however, whether the energy-adjusted Nutri-Score provides a similar or better classification compared with such profiles has not yet been tested. Introducing new algorithms for specific food categories creates challenges since it is difficult to define clear, reasonable food categories, and the scoring might no longer be fair across categories and for mixed products and ready meals. Also, the scoring will be more complex for the users to calculate.

A further theoretical consideration concerns the close relationship between energy density and several of the nutrients already included in the Nutri-Score algorithm. Energy density is not an independent food characteristic, but is largely determined by the content of macronutrients, particularly fat, sugars, and to some extent protein. These nutrients are already incorporated into the Nutri-Score algorithm. Consequently, adjusting nutrient scores according to energy density introduces a degree of conceptual overlap between the weighting variable and the variables being weighted. This overlap complicates the use of energy-density-based weighting because the adjustment may partly re-weight nutritional characteristics that are already represented in the scoring system. In practice, foods with high energy density receive lower weighting factors, while foods with low energy density receive higher weighting factors. Since energy density itself is strongly influenced by nutrients already included in the Nutri-Score algorithm, the weighting process may amplify or attenuate the influence of these nutrients beyond their intended contribution to the final score. This conceptual circularity may help explain some of the unintended scoring outcomes observed in the present study. Several high-energy-density food categories obtained more favourable classifications with the energy-adjusted algorithm. One possible explanation is that lowering the weighting of nutrient components in high-energy-density foods effectively reduced the influence of unfavourable nutrients already contributing to their high energy density. Conversely, low-energy-density foods received greater weighting of their nutrient content, resulting in a stronger impact of both favourable and unfavourable nutrients on the final score. Thus, although energy-density adjustment was intended to compensate for potential distortions associated with scoring per 100 g, it may also have introduced new distortions arising from the non-independence between energy density and the nutritional characteristics already captured by the Nutri-Score algorithm. We did attempt to exclude energy as an indicator from the energy-adjusted algorithm, but this gave even more favourable scores to products with high energy density such as snacks and desserts. Future research could explore alternative approaches for addressing differences in energy density while minimizing conceptual overlap with the nutrients already included in the nutrient profiling model.

The objective of the current study was to perform a proof-of-concept test for adapting an across-the-board model to different levels of energy density rather than develop a fully optimised algorithm. Therefore, we evaluated a single weighting structure designed to balance nutrient content per 100 g and per 100 kcal.The final weighting factors were selected empirically and were not derived through formal optimisation. During preliminary testing, extreme weighting factors resulted in implausible scoring outcomes and were therefore capped, resulting in the final four-level weighting structure. We did not systematically evaluate alternative weighting schemes or perform sensitivity analyses to assess the robustness of the selected weighting factors. Consequently, it cannot be excluded that alternative weighting approaches may have reduced some of the undesired effects observed in the present study. In addition, the total points given for each of the nutritional components were not adjusted in the modified algorithm, but modifications in points might have helped to improve the scoring. A further methodological consideration concerns the recalibration of the Nutri-Score A-E classification thresholds. The energy-adjusted weighting system systematically shifted the distribution of total scores towards less favourable values. Without recalibration, a substantially larger proportion of products would have been assigned poorer Nutri-Score categories compared with the Nutri-Score 2023. The thresholds were therefore recalibrated with the aim of maintaining a distribution of products across Nutri-Score classes that was broadly comparable to that observed with the original algorithm, thereby enabling a more meaningful comparison of classifications between the two systems. The revised thresholds were derived empirically from the score distribution in the study dataset after implementation of the energy-adjusted weighting scheme. We did not evaluate alternative approaches, such as retaining the Nutri-Score 2023 thresholds or testing multiple threshold calibration strategies, as the objective of this study was to perform a proof-of-concept assessment of the proposed methodology rather than to develop a fully optimised classification system. Consequently, both the weighting scheme and the classification thresholds were changed simultaneously. An important implication is that the independent effects of the energy-density weighting and the threshold recalibration cannot be disentangled within the current study design. Therefore, the observed differences between the Nutri-Score 2023 and the energy-adjusted version should be interpreted as the combined effect of these two modifications rather than being attributable exclusively to the weighting system itself.

Beyond its application to nutrient profiling, the proposed methodology with adapting an across-the-board model to different levels of energy density may also be useful in other settings, beyond scoring foods by nutritional quality. This methodology could potentially be relevant in the science of scoring food based on environmental footprints. Although we did not evaluate environmental indicators, several of the methodological elements we examined, such as the choice of reference unit (per 100 g vs. per 100 kcal), weighting, and capping, are also central in nutritional life cycle assessments and in emerging environmental profile models that aim to combine nutrition and environmental indicators for food labelling. This suggests a potential conceptual transferability of the approach, but empirical testing remains for future work.

Validation is an important step when developing new nutrient profile models [20]. Comprehensive validation may include comparison with expert rankings of foods, consistency with national or international dietary guidelines, associations with established diet-quality indices, and comparison with independent classifications of food healthfulness [20]. In addition, consumer-oriented outcomes, such as understanding of the scoring system and purchasing behaviour [21], and health outcomes [22] may provide valuable evidence of the practical relevance of a nutrient profiling model. While thorough testing is required to confirm the validation of a nutrient profile, we only tested the convergent validity against the Nutri-Score 2023. A specific goal of the algorithm was to avoid major changes in scores for foods other than ready meals. However, this was not achieved. Therefore, no further validation of this energy-adjusted version was considered necessary. Consequently, the present findings should be interpreted as a proof-of-concept evaluation rather than a comprehensive validation of a new nutrient profiling system. In its current form, the energy adjusted version of the Nutri-Score is not superior to the Nutri-Score 2023 in classifying foods by nutritional quality across-the-board as it creates too many unwanted side-effects.

A strength of this study was the use of two large food databases comprising most pre-packed foods on the Norwegian market. A limitation was the lack of data on fibre and content of FVL among the products in the database, which may have introduced classification bias. Foods with a high carbohydrate content (such as bread and other grains) mostly had data on fibre, but the content of both fibre and FVL is likely underestimated in some ready meals. Thus, some ready meals may have obtained a poorer Nutri-Score due to this. However, as the same fibre and FVL assumptions were applied in both the Nutri-Score 2023 and the energy-density-adjusted algorithms, the relative comparison between the two algorithms is likely less affected than the absolute Nutri-Score classifications. Nevertheless, some of the observed shifts in scores may have been caused by misclassification. The study relies on Norwegian food databases, which may limit the generalizability of the findings to other regions with different dietary habits and food compositions. Another limitation of this study was that we tested only one set of weighting factors. Each additional weighting scheme would require a full evaluation of score distributions, cross-classification, food category impacts, and unintended consequences for more than 25,000 food items, essentially repeating the entire analytical workflow, which was not feasible in our project. However, regardless of the weight given to the content of components relative to energy density, the same effects and side-effects are likely to occur also if using other weighting factors, although to a different extent. Furthermore, because the weighting scheme and the A-E classification thresholds were modified simultaneously, the contribution of each modification to the observed changes in Nutri-Score classifications could not be assessed separately.

## Conclusion

While the proposed energy-adjusted Nutri-Score algorithm enhanced variation in scoring for certain low-energy-density food categories, such as ready meals, it also exhibited inconsistencies and weaknesses. Low-fat alternatives were penalized more for unfavourable components like salt, causing the scoring to no longer consistently favour products with lower energy and saturated fat content. Further, high-energy-density food categories, such as biscuits, cakes and pastries and sweet snacks and desserts, more frequently received favourable Nutri-Scores, indicating that the energy-adjusted Nutri-Score did not appear superior to the Nutri-Score 2023. Further refinement of the algorithm could address some of the observed side-effects. The methodology itself, adapting an across-the-board nutrient profiling model to energy density, may still hold potential for being a useful approach in a different setting beyond nutrient profiling, for example, in scoring foods based on environmental footprint.

## Data Availability

The data that support the findings of this study are available from Tradesolution and NorgesGruppen (Unil), but restrictions apply to the availability of these data, which were used under license for the current study, and so are not publicly available. Data may however be available from the authors upon reasonable request and with the permission of Tradesolution and NorgesGruppen. Data from Tradesolution is publicly available from vetduat.no.

## Declarations

### Generative AI and AI-assisted technologies

During the preparation of this work, the authors used GPT UiO in order to clarify the text in the Methods section. After using this tool/service, the authors reviewed and edited the content as needed and takes full responsibility for the content of the published article.
